# Behavioural and nutritional risk factors for cardiovascular diseases among the Ghanaian population- a cross-sectional study

**DOI:** 10.1186/s12889-024-17709-5

**Published:** 2024-01-16

**Authors:** Francis Agyekum, Aba Ankomaba Folson, Benjamin Abaidoo, Lambert Tetteh Appiah, Yaw Adu-Boakye, Harold Ayetey, Isaac Kofi Owusu

**Affiliations:** 1 Department of Medicine, College of Health Sciences, University of Ghana Medical School, University of Ghana, Accra, Ghana; 2 Department of Medicine, University of Health and Allied Sciences, Ho, Volta Region Ghana; 3 Department of Surgery, College of Health Sciences, University of Ghana Medical School, University of Ghana, Accra, Ghana; 4 Department of Medicine, School of Medicine and Dentistry, College of Health Sciences, Kwame Nkrumah University of Science and Technology, Kumasi, Ghana; 5 Department of Medicine, School of Medical Sciences, University of Cape Coast, Cape Coast, Ghana

**Keywords:** Physical inactivity, Nutritional risk factors, Smoking, Cardiovascular diseases, Behavioural risk factors, Lifestyle risk factors

## Abstract

**Background:**

Lifestyle behavioural risk factors have been linked to increased cardiovascular disease. Recent data have shown increased atherosclerotic cardiovascular disease (ASCVD) burden in Ghana. This study aimed to describe the behavioural and nutritional risk factors for ASCVD among Ghanaians, and how these risk factors vary by ethnicity, demography and residence.

**Methods:**

We used data from the Ghana Heart Study, a community-based cross-sectional study that recruited participants from eight communities from four regions using a multi-stage sampling technique. Information about various lifestyle behaviours (LBs), including cigarette smoking, alcohol intake, physical inactivity, and fruit and vegetable intake, was obtained using a questionnaire. Data was analysed using IBM SPSS statistics 25. Univariate and multivariate analysis was used to test associations between demographic characteristics and various LBs.

**Results:**

The participants' median (interquartile) age was 46.0 (27.0) years. Of the 1,106 participants (58% females, 80.4% urban dwellers), 8.6% reported using tobacco, 48.9% alcohol, 83.7% physically inactive, 81.4% and 84.9% inadequate fruit and vegetable intake, respectively. Age, sex, ethnicity, and religion were associated with tobacco use, whereas age, sex, educational level, marital status, ethnicity, employment status, and region of residence were associated with physical inactivity. Similarly, ethnicity, employment status, and residence region were associated with inadequate fruit and vegetable intake. Rural dwellers were more likely to be physically inactive and consume inadequate fruits and vegetables. Almost 92% had a combination of two or more LBs. The main predictors of two or more LBs for ASCVD were educational level, marital status, ethnicity, and employment status.

**Conclusion:**

Lifestyle risk factors for ASCVD were highly prevalent in Ghana, with significant age, sex, ethnic, and regional differences. These risky lifestyle behaviors tend to occur together and must be considered in tailoring public health education.

**Trial registration:**

The study was registered at http://www.chictr.org.cn as ChiCTR1800017374.

## Introduction

Atherosclerotic cardiovascular disease (ASCVD) is the leading cause of mortality globally [1]. Sadly, low- and middle-income countries carry over three-quarters (85%) of this mortality burden [2]. In Ghana, ASCVDs are common and mainly driven by strokes [3–5] and coronary artery disease which contributes significantly to the burden of heart failure [6–8]. Several risk factors have been identified in previous studies contributing to the high burden of stroke and other ASCVDs in Ghana and Nigeria including hypertension, dyslipidaemia, regular meat intake, central obesity, cigarette smoking, and diabetes mellitus [9, 10]. Others include inadequate fruit and vegetable intake, physical inactivity, and psychosocial stress.

Modifiable risk factors are responsible for over 90% of the ASCVD burden [11, 12]. These can be classified as behavioural (smoking, physical inactivity, excessive alcohol use), nutritional (unhealthy eating habits), and metabolic (obesity, hypertension, type 2 diabetes mellitus, insulin resistance, dyslipidaemia, etc.) risk factors. The behavioural and nutritional risk factors together are termed lifestyle behaviours (LBs). A variety of LBs play a significant role in developing ASCVDs. These have been linked to the development of cardiometabolic risk factors which increase the risk of atherosclerosis in addition to other mechanisms such as their effects on inflammatory and nutrient sensing pathways, endocrine signalling, autonomic function, body composition, intestinal microbiome, and autophagy [13]. Furthermore, marked differences in the lifetime risk of ASCVDs have been reported across different age spectra, sex, ethnic and birth cohort, which are related to differences in risk-factor burden [14].

Ghana’s epidemiologic and economic transition has resulted in dietary transitions towards more energy-dense and low vegetable intake, as well as more sedentarism and other LBs [15]. In the Ghana Heart Study, we have identified the main cardiometabolic risk factors in Ghana as dyslipidaemia (34.4%), hypertension (26.1%), obesity (15.1%), hyperuricaemia (9.3%), and diabetes mellitus (6.8%) with the main LBs being alcohol use, physical inactivity, and inadequate fruit and vegetable intake [16]. However, these risk factors, though described may show some regional/ethnic differences which may help in planning out public health interventions. Characterizing these LBs regarding ethnicity, demographic differences, and residence might offer policymakers and health planners the necessary information for developing more targeted health promotion and prevention strategies against the rising burden of ASCVDs in Ghana. This study aimed to describe the behavioural and nutritional risk factors for ASCVD among Ghanaians, and how these risk factors vary by ethnicity, demographic factors, and residence.

## Methods and materials

### Study design

This was part of the Ghana Heart Study, the detailed methodology of which has previously been described [16]. The study was registered at http://www.chictr.org.cn as ChiCTR1800017374.

It was a nationwide cross-sectional study using a 3-stage stratified random sampling strategy to recruit participants from 8 communities (4 urban and 4 rural) from four demographically different regions in Ghana between September 2016 and March 2017. One rural and one urban community were selected from Ashanti, Greater Accra, Central, and Northern regions of Ghana by simple random sampling. In each community, a systematic sampling technique was used to select households. Three participants from each household were selected by simple random sampling. Household members who were below 18 years, pregnant, had type 1 diabetes mellitus, secondary hypertension, congenital heart disease, or refused consent were excluded. In addition, members who had self-reported history of ASCVD (personal history of stroke, “heart attack” or peripheral artery disease) were excluded from the whole study.

### Data collection and measurements

Participants were invited to a central location (schools or churches within the communities) for data collection. The demographic information of all the participants was obtained using a standard questionnaire which was completed by trained research assistants who were Medical Officers and nurses. This information included age, date of birth, sex, ethnicity, smoking history, alcohol history, personal and family history of medical illness, and exercise and nutritional history. Age was classified as < 40 (young adult), 40–59 (mid age adult), and > / = 60 (old adult) following the study on the classification of age groups based on facial features by Wen-Bing Horng et al. [17] Smoking was defined as the use of any tobacco products such as cigarettes, cigars, and pipes, either daily or occasionally. Adequate physical activity was defined as regular moderate physical activity (brisk walking, jogging, cycling, swimming, dancing, gardening) for at least 150 min per week [18]. The information on physical activity level was obtained by participant self-report. Nutritional history about fruit and vegetable intake was obtained by dietary propensity questionnaire using the WHO STEPS instrument core questions on diet [19, 20]. Adequate fruit intake was defined as taking at least 2 servings of fruit per day. Adequate vegetable intake was defined as taking at least two servings of vegetable per day [16, 18]. Alcohol history was obtained from the participants about the quantity and type of alcohol used. These were then converted to units per week by the trained research assistants. Excessive alcohol intake was defined as more than 7 units of alcohol per week in women or greater than 14 units per week in males [18]. A few participants who refused consent to participate in the study were replaced by other family members.

### Data handling and ethical issues

Strict confidentiality was always maintained. This was achieved using a coded questionnaire. Ethical approval for this study (CHRPE/AP/415/16) was provided by the Committee on Human Research, Publications and Ethics of the Kwame Nkrumah University of Science and Technology and the Komfo Anokye Teaching Hospital, Kumasi, Ghana. A written informed consent was obtained from all participants before inclusion. The study was conducted in strict adherence to the protocol. Recruitment into the study began from September 2016 and ended in March 2017. The authors vouch for the fidelity of the data. All methods were carried out in accordance with the Helsinki Declaration.

### Statistical analysis

The statistical analysis was done with IBM SPSS version 25 statistical software. Test for normality of the data was done with the Kolmogorov–Smirnov test. Normally distributed variables were analyzed and presented as mean and standard deviations and non-normally distributed variables were presented as median (interquartile range) values. Simple proportions were used to summarize the prevalence of behavioral and nutritional risk factors such as physical inactivity, cigarette smoking, excessive alcohol intake, and inadequate fruit and vegetable intake.

A univariate analysis was used in analyzing the associations between socio-demographic characteristics and risk factors for ASCVD. Multicollinearity in the model was accessed in the dependent variables and the independent variables. Where the Variance Inflation factor (VIF) is less than or equal to 10 then no multicollinearity exists in the model. Further to this analysis, a multivariate logistic regression analysis was used to assess the associations between the socio-demographic characteristics and risk factors for developing cardiovascular diseases for all variables with significant association in the univariate model. Odds ratios and 95% confidence intervals were presented and p-value less than 0.05 were described as statistically significant. Additionally, a dichotomized variable was created as an outcome variable for participants with multiple risk factors for developing cardiovascular diseases and those with no or a single risk factor. A multivariate logistic regression analysis of two or more behavioral and nutritional risk factors for cardiovascular diseases was done. Additionally, combinations of risk factors occurring together most frequently and frequencies of having 1, 2, 3, 4, or more risk factors were assessed and documented.

## Results

### General characteristics and prevalence of behavioral and nutritional risk factors among participants

A total of 1,106 Ghanaian adults participated in this study. The median (interquartile) age was 46.0 (27.0) years. The minimum age was 18 years, and the maximum was 95 years (Table 1). A total of 436 (39.4%) participants were under 40 years. There was a female preponderance (642, 58.0%). The majority (858, 77.6%) had a low level (educational level below tertiary education) of education. More than half (588, 53.2%) of the participants were married and more than half (639, 57.8%) were Akans. Majority (686, 62.0%) were employed. Participants from the Ashanti region were more (353, 31.9%) than those from the other regions. Urban dwellers were the majority (889, 80.4%). A total of 388 (35.1%) and 103 (9.3%) were hypertensive and diabetic respectively (Table 1).

**Table 1:** General characteristics of the study population

Demographics	Total	Behavioural risk factors			Nutritional risk factors	
	N(%) 1106(100.0)	Tobacco use N(%) 95(8.6)	Alcohol use N(%) 541(48.9)	Physical inactivity N(%) 926(83.7)	Inadequate fruit intake N(%) 900(81.4)	Inadequate vegetable intake N(%) 939(84.9)
Age (years)						
< 40	436(39.4)	25(2.3)	213(19.3)	367(33.2)	366(33.1)	375(33.9)
40–59	382(34.5)	41(3.7)	183(16.5)	299(27.0)	305(27.6)	320(28.9)
> = 60	288(26.0)	29(2.6)	145(13.1)	260(23.5)	229(20.7)	244(22.1)
Sex						
Male	464(42.0)	80(7.2)	262(23.7)	363(32.8)	374(33.8)	385(34.8)
Female	642(58.0)	15(1.4)	279(25.2)	563(50.9)	526(47.6)	554(50.1)
Education						
High	248(22.4)	19(1.7)	95(8.6)	168(15.2)	161(14.6)	165(14.9)
Low	858(77.6)	76(6.9)	446(40.3)	758(68.5)	739(66.8)	774(70.0)
Marital status						
Married	588(53.2)	59(5.3)	297(26.9)	512(46.3)	517(46.7)	540(48.8)
Separated	113(10.2)	7(0.6)	31(2.8)	38(3.4)	34(3.1)	38(3.4)
Single	405(36.6)	29(2.6)	213(19.3)	376(34.0)	349(39.6)	361(32.6)
Ethnicity						
Akan	639(57.8)	62(5.3)	396(35.8)	582(52.6)	560(50.6)	592(53.5)
Dagomba	148(13.4)	7(0.6)	4(0.4)	133(12.0)	133(12.0)	145(13.1)
Ga	93(8.4)	16(1.4)	69(6.3)	75(6.8)	76(6.9)	76(6.9)
Ewe	56(5.1)	4(0.4)	31(2.8)	44(4.0)	44(4.0)	45(4.1)
Hausa	12(1.1)	1(0.1)	2(0.2)	11(1.0)	8(0.7)	4(0.4)
Others	158(14.3)	5(0.5)	39(3.5)	81(7.3)	79(7.1)	77(7.0)
Employment						
Employed	686(62.0)	59(5.3)	368(33.3)	594(53.7)	600(54.2)	631(57.1)
Unemployed	420(38.0)	36(3.3)	173(15.6)	332(30.0)	300(27.1)	308(27.8)
Region						
Accra	352(31.8)	35(3.2)	232(21.0)	300(27.1)	273(24.7)	273(24.7)
Ashanti	353(31.9)	39(3.5)	193(17.5)	325(28.5)	310(28.0)	329(29.7)
Central	197(17.8)	14(1.3)	114(10.3)	159(14.4)	166(15.0)	174(15.7)
Northern	204(18.4)	7(0.6)	2(0.2)	152(13.7)	151(13.7)	163(14.7)
Residence						
Rural	217(19.6)	22(2.0)	111(10.0)	156(14.1)	170(15.4)	181(16.4)
Urban	889(80.4)	73(6.6)	430(38.9)	770(69.6)	730(66.0)	758(68.5)
Diabetes Mellitus	103(9.3)	6(0.5)	59(5.3)	92(8.3)	89(8.0)	92(8.3)
Hypertension	388(35.1)	44(4.0)	212(19.2)	341(30.8)	331(29.9)	343(31.0)

Median (interquartile) age = 46.0 (27) years. Minimum age = 18 years and maximum age = 95 years. Other (minor) ethnic groups with total numbers below ten included; Kusasi (9, 0.8%), Guans (9, 0.8%), Gurma (9, 0.8%), Guan (9, 0.8%), Grusi (9, 0.8%), Mande (9, 0.8%), Gonja (9, 0.8%), Gurunsi (9, 0.8%), Nzema (9, 0.8%), Kyode (8, 0.7%), Mamprusi (8, 0.7%), Mossi(8, 0.7%), Soninke (8, 0.7%), Wala (8, 0.7%), Tabom (8, 0.7%), Tallensi (8, 0.7%), Ahanta (8, 0.7%), Assin (8, 0.7%), and Nanumba (5, 0.4%) respectively

The prevalence of behavioural and nutritional risk factors for ASCVDs are presented in Table 1. A total of 8.6% of the participants reported the use of tobacco, 48.9% used alcohol, and 83.7% were physically inactive. The majority (81.4%, 84.9%) of the participants recorded inadequate fruit and vegetable intake respectively (Table 1).

### Univariate analysis of behavioral and nutritional risk factors for ASCVD among Ghanaian adults

From the univariate analysis, age was associated with tobacco use and physical inactivity respectively (*p* < 0.005). Sex was also associated with the use of tobacco, alcohol, and physical inactivity (*p*-values < 0.05) (Table 2). Education was associated with the use of alcohol, physical inactivity, inadequate fruit intake, and inadequate vegetable intake (*p* < 0.05).

**Table 2:** Univariate analysis of socio-demographic characteristics and behavioural and nutritional risk factors for ASCVD

Demographics	Behavioural risk factors OR (95% CI); *P*-value			Nutritional risk factors	
	Tobacco use *N* = 95	Alcohol use *N* = 541	Physical inactivity *N* = 926	Inadequate fruit intake *N* = 900	Inadequate vegetable intake *N* = 939
Age (years)					
< 40	1.8(1.1–3.2); 0.032*	1.1(0.8–1.4);0.694	1.7(1.1–2.8);0.019*	0.7(0.5–1.1);0.128	0.9(0.6–1.4);0.630
40–59	0.9(0.6–1.5); 0.781	1.1(0.8–1.5);0.531	2.6(1.6–4.1);0.001*	1.0(0.7–1.4);0.917	1.1(0.7–1.6);0.738
> = 60	Reference	Reference	Reference	Reference	Reference
Sex					
Male	8.7(4.9–15.3);0.001*	1.7(1.3–2.1);0.01*	0.5(0.4–0.7);0.001*	0.9(0.7–1.2);0.585	0.8(0.6–1.1);0.148
Female	Reference	Reference	Reference	Reference	Reference
Education					
High	0.9(0.5–1.4);0.609	0.6(0.4–0.8);0.01*	0.3(0.2–0.4);0.001*	0.3(0.2–0.4);0.01*	0.2(0.1–0.3);0.01*
Low	Reference	Reference	Reference	Reference	Reference
Marital status					
Married	0.7(0.4–1.1);0.119	1.1(0.8–1.4);0.519	1.9(1.2–3.0);0.004*	0.9(0.6–1.2);0.417	0.7(0.4–1.1);0.150
Separated	1.2(0.5–2.4); 0.721	2.9(1.9–4.6);0.001*	25.6(14.9–44.1);0.01*	14.5(8.9–23.7);0.01*	16.1(9.8–26.7);0.01*
Single	Reference	Reference	Reference	Reference	Reference
Ethnicity					
Akan	0.3(0.1–0.8);0.012*	0.2(0.1–0.3);0.001*	0.1(0.1–0.2);0.001*	0.1(0.1–0.2);0.01*	0.1(0.04–0.12);0.01*
Dagomba	0.7(0.2–2.1);0.484	0.8(0.1–1.9);0.01*	0.1(0.1–0.2);0.001*	0.1(0.1–0.2);0.01*	0.02(0.01–0.06);0.01*
Ga	0.2(0.1–0.4);0.001*	0.1(0.1–0.2);0.001*	0.3(0.1–0.5);0.001*	0.2(0.1–0.4);0.01*	0.2(0.1–0.4);0.01*
Ewe	0.4(0.1–1.6);0.215	0.3(0.1–0.5);0.001*	0.3(0.1–0.6);0.001*	0.3(0.1–0.6);0.01*	0.2(0.1–0.5);0.01*
Hausa	0.4(0.1–3.4);0.369	1.6(0.3–7.8);0.535	0.1(0.1–0.8);0.026*	0.5(0.1–1.7);0.27	1.9(0.6–6.6);0.01*
Others	Reference	Reference	Reference	Reference	Reference
Employment					
Employed	1.0(0.7–1.5);0.540	1.6(1.3–2.1);0.001*	1.7(1.2–2.4);0.001*	2.8(2.0–3.8);0.01*	4.2(2.9–5.9);0.01*
Unemployed	Reference	Reference	Reference	Reference	Reference
Region					
Accra	0.3(0.1–0.7);0.007*	0.01(0.001–0.02);0.01*	0.5(0.3–0.8);0.002*	0.8(0.6–1.2);0.345	1.2(0.8–1.8);0.517
Ashanti	0.3(0.1–0.7);0.003*	0.01(0.002–0.03);0.01*	0.4(0.2–0.6);0.001*	0.4(0.3–0.6);0.001*	0.3(0.2–0.5);0.01*
Central	0.5(0.2–1.2);0.464	0.01(0.002–0.03);0.01*	0.7(0.4–1.1);0.138	0.5(0.3–0.9);0.012*	0.5(0.3–0.9);0.023*
Northern	Reference	Reference	Reference	Reference	Reference
Residence					
Rural	1.3(0.8–2.1);0.347	1.1(0.8–1.5);0.496	0.4(0.3–0.6);0.001*	0.8(0.5–1.1);0.207	0.9(0.6–1.3);0.526
Urban	Reference	Reference	Reference	Reference	Reference

^*^Significant risk factor

Marital status was associated with alcohol use, physical inactivity, inadequate fruit and vegetable intake respectively (*p* < 0.05).

Ethnicity was associated with tobacco use, alcohol use, physical inactivity, inadequate fruit and vegetable intake (*p* < 0.05).

Employment was associated with alcohol use, physical inactivity, inadequate fruit and vegetable intake (*p* < 0.05).

Region of residence was also associated with tobacco and alcohol use, physical inactivity, and inadequate fruit and vegetable intake (*p* < 0.05). Residency was associated with physical inactivity (*p* < 0.05) (Table 2).

### Multivariate analysis of nutritional risk factors for ASCVD among Ghanaian adults

From the multivariate analysis, participants aged below 40 years were about 2 times more likely to use alcohol compared to participants aged 60 years and above (*p* = 0.016). Using participants aged 60 years and above as reference, participants below 40 years and those 40–59 years were about 3 times and 4 times more likely to be physically inactive respectively (*p* < 0.005) (Table 3).

**Table 3:** Multivariate analysis of socio-demographic characteristics and behavioural and nutritional risk factors for cardiovascular diseases

Demographics	Behavioural risk factors AOR (95% CI); *P*-value			Nutritional risk factors	
	Tobacco use *N* = 95	Alcohol use *N* = 541	Physical inactivity *N* = 926	Inadequate fruit intake *N* = 900	Inadequate vegetable intake *N* = 939
Age (years)					
< 40	1.8(0.9–3.4); 0.076	1.6(1.1–2.3);0.016*	2.5(1.3–4.7);0.004*	0.7(0.4–1.1);0.151	1.0(0.6–1.8);0.989
40–59	0.7(0.4–1.4); 0.335	1.4(0.9–2.1);0.124	3.7(1.9–6.9);0.001*	1.1(0.7–1.8);0.667	1.6(0.9–3.1);0.117
> = 60	Reference	Reference	Reference	Reference	Reference
Sex					
Male	10.5(5.8–18.9);0.001*	2.5(1.9–3.4);0.01*	0.4(0.2–0.6);0.001*	0.9(0.8–1.4);0.880	1.2(0.7–1.8);0.512
Female	Reference	Reference	Reference	Reference	Reference
Education					
High	1.2(0.7–2.2);0.549	1.6(1.1–2.4);0.014*	1.3(0.8–2.1);0.375	1.5(0.9–2.4);0.083*	1.8(1.0–3.0);0.041*
Low	Reference	Reference	Reference	Reference	Reference
Marital status					
Married	0.8(0.5–1.4); 0.498	0.9(0.7–1.3);0.657	1.5(0.9–2.5);0.128	0.8(0.5–1.2);0.310	0.7(0.4–1.2);0.223
Separated	0.8(0.3–2.2); 0.624	1.5(0.9–2.7);0.153	22.4(10.9–45.2);0.01*	9.9(5.4–18.2);0.001*	11.7(5.8–23.8);0.01*
Single	Reference	Reference	Reference	Reference	Reference
Ethnicity					
Akan	0.3(0.1–1.0);0.056	0.5(0.3–0.8);0.009*	0.3(0.2–0.6);0.001*	0.6(0.3–1.0);0.051	0.3(0.2–0.6);0.01*
Dagomba	0.1(0.01–1.3);0.083	1.4(0.3–5.9);0.683	0.2(0.1–0.6);0.001*	0.3(0.1–0.7);0.006*	0.1(0.02–0.3);0.01*
Ga	0.2(0.1–0.6);0.006*	0.4(0.2–0.8);0.012*	0.4(0.2–1.1);0.070*	0.4(0.2–0.8);0.014*	0.3(0.1–0.7);0.006*
Ewe	0.4(0.1–2.0);0.274	0.9(0.4–1.8);0.736	0.5(0.2–1.3);0.136	0.6(0.3–1.5);0.281	0.5(0.2–1.2);0.100
Hausa	0.3(0.02–3.3);0.312	5.4(1.1–28.0);0.044*	0.3(0.04–2.9);0.316	1.4(0.4–5.2);0.634	5.6(1.4–22.6);0.015*
Others	Reference	Reference	Reference	Reference	Reference
Employment					
Employed	1.3(0.7–2.3);0.363	0.6(0.5–0.9);0.008*	0.9(0.6–1.6);0.840	0.6(0.4–0.9);0.022*	0.3(0.2–0.6);0.01*
Unemployed	Reference	Reference	Reference	Reference	Reference
Region					
Accra	0.1(0.014–1.3);0.087	0.01(0.001–0.05);0.01*	0.5(0.2–1.3);0.149	1.1(0.5–2.4);0.861	2.0(0.8–5.1);0.126
Ashanti	0.1(0.01–0.8);0.035*	0.01(0.002–0.08);0.01*	0.2(0.1–0.6);0.002*	0.3(0.1–0.6);0.003*	0.2(0.1–0.5);0.01*
Central	0.1(0.01–1.4);0.092	0.01(0.002–0.07);0.01*	0.9(0.4–2.4);0.936	0.5(0.2–1.3);0.145*	0.6(0.2–1.7);0.321
Northern	Reference	Reference	Reference	Reference	Reference
Residence					
Rural	0.9(0.5–1.5);0.640	1.3(0.9–1.9);0.189	6.3(3.8–10.3);0.001*	2.1(1.3–3.4);0.002*	2.1(1.2–3.8);0.010*
Urban	Reference	Reference	Reference	Reference	Reference

^*^Significant risk factor

Males were about 11 times and 3 times more likely to use tobacco and alcohol compared to females respectively. Males were less likely to be physically inactive compared to females (AOR 0.4, *p* = 0.001) (Table 3).

Using participants with low education as reference, participants with high education were 2 times more likely to use alcohol and have inadequate fruit and vegetable intake respectively (*p* < 0.05).

Participants who are separated in marriage were about 22 times, 10 times, and 12 times more likely to be physically inactive, consume inadequate fruits and vegetables respectively (*p* < 0.05).

Referring to other smaller ethnic groups, Ga participants were less likely to use tobacco (AOR = 0.2, *p* < 0.05). Akan, and Ga participants were less likely to use alcohol (AOR 0.5 and 0.4 respectively, *p* < 0.05), whilst Hausas were about 5 times more likely to use alcohol compared to the other ethnic groups (*p* < 0.05). Akans, Dagombas, and Gas were less likely to be physically inactive. Dagombas and Gas were less likely to consume inadequate fruit. Akans, Dagombas, and Gas were less likely to consume inadequate vegetables respectively compared to the other tribes (AOR 0.3, 0.1, 0.3, respectively, *p* < 0.05). Hausas were 6 times more likely to have inadequate vegetables compared to the other tribes (*p* < 0.05).

Employed participants were less likely to use alcohol, and consume inadequate fruits and vegetables compared to unemployed participants (AOR = 0.6, 0.6, 0.3 respectively, *p* < 0.05).

Participants from the Ashanti region were less likely to use tobacco (AOR = 0.1, *p* < 0.05). Participants from Accra, Ashanti, and Central were less likely to use alcohol compared to the Northern region (AOR = 0.01, *p* < 0.05). Participants from Ashanti region were less likely to be physically inactive, and consume inadequate fruit and vegetables compared to the Northern region (AOR = 0.2, *p* < 0.01). Similarly, rural folks were 6.3, 2.1, and 2.1 times more likely to be physically inactive, consume inadequate fruits and vegetables respectively, compared to urban folks (*p* < 0.05) (Table 3).

### Multivariate analysis of two or more behavioural and nutritional risk factors for ASCVD among Ghanaian adults

In a multivariate analysis of two or more behavioural and nutritional risk factors for ASCVD, the predictors were; education, marital status, ethnicity, and employment status. Participants with higher educational level were less likely to have two or more behavioural and nutritional risk factors for cardiovascular diseases compared to those with lower educational level [0.3(0.1–0.7); 0.010]. Compared to singles, married participants were seventeen times more likely to have two or more behavioural and nutritional risk factors for ASCVDs [17.8(6.2–50.9); 0.001]. Ewes and Hausas were nineteen and six times respectively more likely to have two or more behavioural and nutritional risk factors for ASCVDs compared to the other tribes [18.9(1.7–208.5); 0.016] and [6.2(1.8–20.7);0.003]. Employed participants were five times more likely to have two or more behavioural and nutritional risk factors for ASCVDs [5.4(1.8–16.2);0.003] compared to unemployed participants (Table 4).

**Table 4:** Multivariate analysis of two or more behavioural and nutritional risk factors for ASCVD among Ghanaian adults

Demographics	Two or more behavioural and nutritional risk factors for ASCVD		
	Number (%)	AOR (95% CI)	*P*-value
Age (years)			
< 40 (young adult)	408(36.9)	1.1(0.5–3.9)	0.466
40–59 (mid age adult)	349(31.6)	0.7(0.2–2.1)	0.524
> = 60 (old adult)	269(24.3)	Reference category	Reference category
Sex			
Male	422(38.2)	0.6(0.2–1.4)	0.205
Female	604(54.6)	Reference category	Reference category
Education			
High	179(16.2)	0.3(0.1–0.7)	0.010*
Low	847(76.6)	Reference category	Reference category
Marital status			
Married	577(52.2)	17.8(6.2–50.9)	0.001*
Separated	47(4.2)	0.3(0.1–1.2)	0.090
Single	402(36.3)	Reference category	Reference category
Ethnicity			
Akan	633(57.2)	0.4(0.0–4.6)	0.453
Dagomba	147(13.3)	1.6(0.2–10.9)	0.635
Ga	91(8.2)	4.0(0.6–25.3)	0.146
Ewe	54(4.9)	18.9(1.7–208.5)	0.016*
Hausa	11(1.0)	6.2(1.8–20.7)	0.003*
Others	90(8.1)	Reference category	Reference category
Employment			
Employed	678(61.3)	5.4(1.8–16.2)	0.003*
Unemployed	348(31.5)	Reference category	Reference category
Region			
Accra	341(30.8)	0.6(0.2–2.4)	0.481
Ashanti	335(30.3)	1.7(0.4–6.4)	0.444
Central	183(16.5)	2.9(0.7–12.4)	0.144
Northern	167(15.1)	Reference category	Reference category
Residence			
Rural	203(18.4)	0.3(0.1–1.1)	0.069
Urban	823(74.4)	Reference category	Reference category

^*^Significant risk factor

### Combination of behavioural and nutritional risk factors for ASCVD

**Fig. 1:**
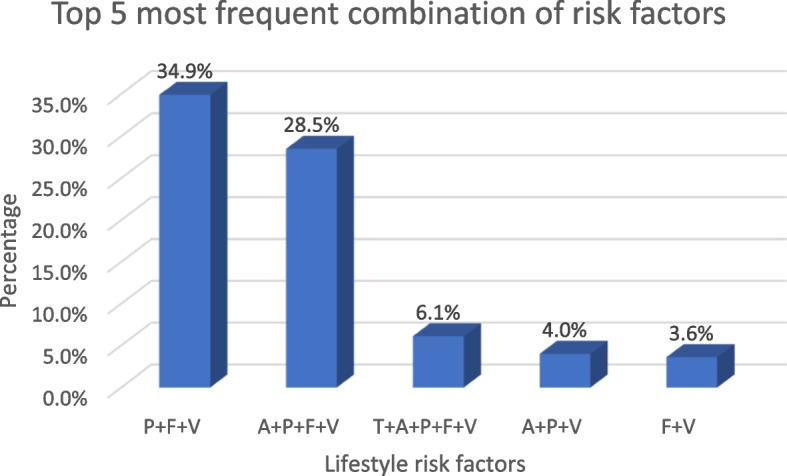
Combination of behavioural and nutritional risk factors for ASCVD. A = Alcohol intake; F = inadequate fruit intake; P = physical inactivity; T = tobacco smoking; V = inadequate vegetables intake

**Fig. 2:**
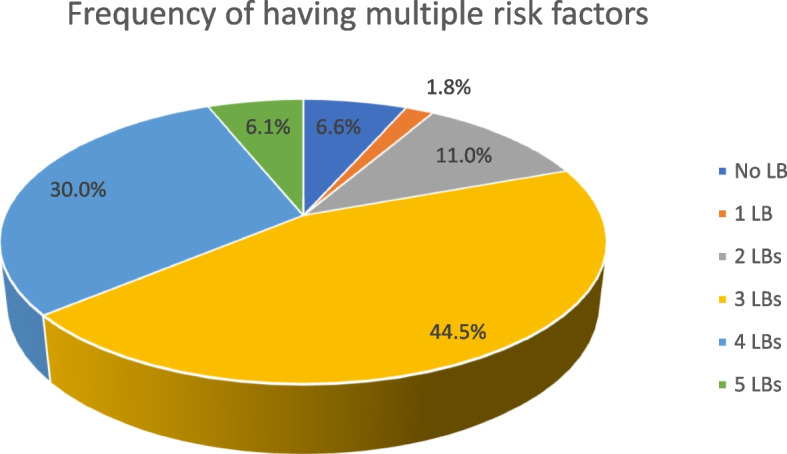
Frequency of having 1,2,3,4 or 5 risk factors. LB = Lifestyle behaviours

Figures 1 and 2 show the combination of behavioural and nutritional risk factors that were present together. A combination of three risk factors was the most frequent (492, 44.5%). The most frequent combination of risk factors for ASCVD was physical inactivity + inadequate fruit intake + inadequate vegetable intake (386, 34.9%).

## Discussion

This study found a high prevalence of behavioural and nutritional risk factors for ASCVDs among the Ghanaian population with significant sex, age, and regional, and ethnic differences. About 85% of the participants reported inadequate vegetable intake, 84% were physically inactive, and 81% reported inadequate fruit intake. Almost half of the participants (about 49%) reported alcohol intake and less than 9% reported smoking tobacco. Many of the participants had multiple lifestyle behavioural risk factors with almost 92% having a combination of two or more risky LBs. The main predictors of two or more LBs for ASCVD were educational level, marital status, ethnicity, and employment status.

The ethnic and regional differences may reflect the influence of cultural differences in food choices, preparation, religion, and gender roles. This may account for some of the regional differences in metabolic risk factors that have been documented in Ghana such as hypertension, [21] diabetes mellitus [22], obesity [23], and dyslipidaemia [24]. Kodaman et. al [25] noted that urban residence was strongly associated with obesity (OR: 7.8, 95% CI: 5.3–11.3), diabetes mellitus (OR 3.6, 95% CI: 2.3–5.7), and hypertension (OR 3.2, 95% CI: 2.6–4.0) in Ghana. Similarly, Tagoe and colleagues, in a previous study, reported that risky LBs were more common in urban areas than rural ones [26]. These nutritional and behavioural differences may also be a major contributor to the ethnic and geographic differences in ASCVDs, in addition to genetic differences [27]. It will be interesting to study whether these lifestyle differences translate into ethnic differences in ASCVD such as stroke and coronary artery disease in Ghana.

The low levels of smoking (8.6%) recorded in this study, with very low rates in women (1.4%) is consistent with previous studies in Ghana [28, 29]. Despite sustained tobacco industry presence in Ghana since the late 1950s; [30] and the economic growth over the years, smoking prevalence has remained low and even decreased [26, 28, 29]. This may be a result of a combination of factors, including health policy, strong media campaigns against smoking, and the positive influence of religion and strong cultural dislike for smoking in Ghana, especially among women [29].

More than four-fifths of the participants in this study were physically inactive. This is similar to the previous findings in Ghana [26]. Other small local studies have supported the low levels of physical activity among specific groups in Ghana [31–35]. It is not apparent why younger people are more likely to be physically inactive compared to those above 60 years. People probably get more time to exercise after retirement at 60 years in Ghana. This is contrary to the findings of a recent scoping review which found most Ghanaians to be physically active. However, this was based on 2000–2008 publications and lacks high-quality nationally representative data [36]. It is an interesting finding that in the multivariate regression analysis, rural dwellers were six times more likely to be physically inactive compared to urban dwellers. This may be related to the availability of fitness clubs and training grounds in the urban areas. A study of 4,425 adult Ghanaian workers found that sedentary work was linked to a higher risk of hypertension compared to moderate physical activity [37].

Close to half of the participants gave a history of regular alcohol consumption with significant sex, regional, and ethnic differences. This may be related to differences in the cultural, religious, and traditional practices among the people in the various regions [38]. The reasons why people of the Hausa tribe, and participants living along the coastal areas were more likely to report alcohol use in this study are not apparent. However, earlier publications have identified myriad factors that drive alcohol usage including positive or negative life events, socio-cultural beliefs, health reasons, spiritual beliefs, and easy access [39]. These findings need further exploration to inform designing specific interventions. Excessive amount of alcohol (> 40 g/day in females and > 60 g/day in males) has been associated with increased CVDs [40, 41]. No randomized controlled trials have confirmed cardiovascular benefits of alcohol. Indeed, risks due to alcohol consumption increase for most cardiovascular diseases, including hypertensive heart disease, cardiomyopathy, atrial fibrillation and flutter, and stroke [42]. More recent publications have indicated that, no amount of alcohol is safe for the heart [43].

Less than 19% and 16% of the participants reported regular intake of fruits and vegetables respectively. In addition to ethnicity and region of residence, marital status, employment status, and educational level were associated with fruit and vegetable intake. The INTERHEART Study showed that lack of daily consumption of fruits and vegetables was associated with increased odds of first myocardial infarction [12]. Similarly, in the SIREN study in West Africa, the dietary factors associated with increased odds of ischaemic stroke included regular meat consumption, low green leafy vegetable consumption, and added salt on the Table [10] The ethnic and regional differences in fruit and vegetable intake may be related to Ghana's cultural differences in food preparation and eating patterns. For example, the finding that Hausas were six times more likely to have inadequate vegetables compared to the other smaller tribes may be related to their traditional means of preparing their meals.

A worrying finding was the propensity of individuals to indulge in multiple unhealthy LBs. More than 90% of the participants had a combination of two or more unhealthy LBs and more than 80% had three or more LBs occurring together. The most common combinations were physical inactivity with inadequate fruit and vegetable intake. The most common four combinations were also these three plus alcohol intake. In a qualitative study, Mensah et al.^39^ intimated that these lifestyle behaviours are driven by five major factors, including economic, medical, psycho-social, sexual, and socio-cultural factors. Any interventions aimed at addressing these LBs should, therefore, consider the factors driving and sustaining them. Additionally, we found that participants with higher education were less likely to have two or more risky LBs for ASCVDs compared to those with lower education. This is similar to the findings of a previous study by Tagoe and colleagues in which having formal education was associated with living healthy, with the chances of making healthy choices increasing with an increasing level of education [26]. Incorporating lifestyle education in the school curriculum at all levels of Ghana’s educational system has likely contributed to these better lifestyle choices. In addition to formal education, strong and consistent media educational campaigns on healthy lifestyle choices are likely to improve outcomes. A recent publication showed that over 71% of Ghanaians had good CVD knowledge and a large proportion of them received their CVD education via radio and television [44]. However, the opposite trend noted with employed people having five times more likelihood to have two or more risky LBs should be a cause for concern. It may suggest that when people are gainfully employed, the psychosocial stress of work demands, together with higher affordability of fast foods and cafeterias causes them to make less healthy choices, potentially dampening the gains from education.

These findings show that policies and interventions should be developed taking the age, sex, regional, and ethnic variations into consideration. The interventions need to be tailored to be culturally, socially, and ethnically acceptable to the people. Furthermore, there should be policies to regulate the advertisement of alcohol on radio and television, while promoting the consumption of fruit and vegetables.

This study has some limitations. The cross-sectional study design does not allow for risk estimation and one can only determine associations. Secondly, the behavioural and nutritional factors were obtained by participant self-report which may suffer from recall bias and interviewer influence. The STEPS core questions on diet may be limited in utility in rural Ghana where families prepare meals together and tend to eat from the same bowl making quantification of individual’s vegetable intake difficult. There was no objective measurement of physical activity levels as well as fruit and vegetable intake. Additionally, the few patients who refused participation and had to be replaced by other family members could introduce some bias into the sampling process. Furthermore, the exclusion of people with established ASCVD from the parent study may have excluded participants with certain characteristics that might have influenced LBs. These limitations notwithstanding, the main strengths of this study are the recruitment of a representative sample from four regions of the country and the use of community level data.

## Conclusions

Risky LBs for ASCVD were highly prevalent in Ghana, with significant age, sex, ethnic, and regional differences. Many people had multiple risky LBs with the most common being physical inactivity, inadequate fruit and vegetable intake, and alcohol use. Higher education was associated with less likelihood of multiple risky LBs whilst employment showed the opposite association. Public health strategies to promote healthy LBs should consider the ethnic, sex, regional, and socio-demographic differences. Further research should also focus on understanding various strategies that are likely to be successful in different contexts.

## Data Availability

The datasets generated and analysed during this study are available from the corresponding author on reasonable request.

## Abbreviations

AOR: Adjusted odds ratio
ASCVD: Atherosclerotic cardiovascular disease
CI: Confidence interval
CVD: Cardiovascular disease
LBs: Lifestyle behaviours
OR: Odds ratio

## References

[1] James SL, Abate D, Abate KH (2018). Global, regional, and national incidence, prevalence, and years lived with disability for 354 diseases and injuries for 195 countries and territories, 1990–2017: a systematic analysis for the Global Burden of Disease Study 2017. The Lancet.

[2] WHO. Cardiovascular Diseases. 11 June 2021 2021. https://www.who.int/news-room/fact-sheets/detail/cardiovascular-diseases-(cvds). Accessed 29 Jan 2023.

[3] Agyekum F, Akumiah F (2023). Atherosclerotic Cardiovascular Disease Burden in Ghana: A Scoping Review. J Clin Prev Cardiol.

[4] Sanuade O, Agyemang C. Stroke in Ghana: a systematic literature review. In de-Graft Aikens A, Agyei-Mensah S, Agyemang C, editors. Chronic Non-communicable Diseases in Ghana: Multidisciplinary Perspectives. Legon, Accra Ghana: Sub-Saharan Publishers. 2013. p. 29–40. (Social Sciences Studies Regional Institute for Population Studies; 1).

[5] Sarfo FS, Mobula LM, Plange-Rhule J, Ansong D, Ofori-Adjei D (2018). Incident stroke among Ghanaians with hypertension and diabetes: A multicenter, prospective cohort study. J Neurol Sci.

[6] Agyekum F, Folson AA, Asare BY-A, et al. Contemporary aetiology of acute heart failure in a teaching hospital in Ghana. BMC Cardiovascular Disorders 2023;23(1):82. 10.1186/s12872-023-03103-3 PMC9921595 36765294

[7] Appiah LT, Sarfo FS, Agyemang C (2017). Current trends in admissions and outcomes of cardiac diseases in Ghana. Clin Cardiol.

[8] Owusu IK, Adu-Boakye Y (2013). Prevalence and aetiology of heart failure in patients seen at a teaching hospital in Ghana. J Cardiovasc Dis Diagn.

[9] Sarfo FS, Ovbiagele B, Akpa O (2022). Risk factor characterization of ischemic stroke subtypes among West Africans. Stroke.

[10] Owolabi MO, Sarfo F, Akinyemi R (2018). Dominant modifiable risk factors for stroke in Ghana and Nigeria (SIREN): a case-control study. Lancet Glob Health.

[11] Flora GD, Nayak MK (2019). A brief review of cardiovascular diseases, associated risk factors and current treatment regimes. Curr Pharm Des.

[12] Yusuf S, Hawken S, Ôunpuu S (2004). Effect of potentially modifiable risk factors associated with myocardial infarction in 52 countries (the INTERHEART study): case-control study. Lancet.

[13] Lechner K, von Schacky C, McKenzie AL (2020). Lifestyle factors and high-risk atherosclerosis: Pathways and mechanisms beyond traditional risk factors. Eur J Prev Cardiol.

[14] Berry JD, Dyer A, Cai X (2012). Lifetime risks of cardiovascular disease. N Engl J Med.

[15] Ecker O, Fang P. Economic development and nutrition transition in Ghana: Taking stock of food consumption patterns and trends. In Achieving a nutrition revolution for Africa: The road to healthier diets and optimal nutrition. Covic N, Hendriks SL, Editors. Chapter 4. Washington, D.C: International Food Policy Research Institute (IFPRI); 2016. pp. 28–50

[16] Li J, Owusu IK, Geng Q (2020). Cardiometabolic risk factors and preclinical target organ damage among adults in Ghana: findings from a national study. J Am Heart Assoc.

[17] Horng W-B, Lee C-P, Chen C-W (2001). Classification of age groups based on facial features. J Appl Sci Eng.

[18] Piepoli MF, Hoes AW, Agewall S (2016). 2016 European Guidelines on cardiovascular disease prevention in clinical practice: The Sixth Joint Task Force of the European Society of Cardiology and Other Societies on Cardiovascular Disease Prevention in Clinical Practice (constituted by representatives of 10 societies and by invited experts)Developed with the special contribution of the European Association for Cardiovascular Prevention & Rehabilitation (EACPR). Eur Heart J.

[19] Riley L, Guthold R, Cowan M (2016). The World Health Organization STEPwise approach to noncommunicable disease risk-factor surveillance: methods, challenges, and opportunities. Am J Public Health.

[20] WHO. Standard STEPS instrument. 1 October 2020 2016. https://www.who.int/publications/m/item/standard-steps-instrument . Accessed Apr 19 2016.

[21] Agyemang C (2006). Rural and urban differences in blood pressure and hypertension in Ghana. West Africa Public Health.

[22] Asamoah-Boaheng M, Sarfo-Kantanka O, Tuffour AB, Eghan B, Mbanya JC (2019). Prevalence and risk factors for diabetes mellitus among adults in Ghana: a systematic review and meta-analysis. Int Health.

[23] Ofori-Asenso R, Agyeman AA, Laar A, Boateng D (2016). Overweight and obesity epidemic in Ghana—a systematic review and meta-analysis. BMC Public Health.

[24] Agongo G, Nonterah EA, Debpuur C (2018). The burden of dyslipidaemia and factors associated with lipid levels among adults in rural northern Ghana: An AWI-Gen sub-study. PLoS ONE.

[25] Kodaman N, Aldrich MC, Sobota R (2016). Cardiovascular disease risk factors in Ghana during the rural-to-urban transition: a cross-sectional study. PLoS ONE.

[26] Tagoe HA, Dake FAA (2011). Healthy lifestyle behaviour among Ghanaian adults in the phase of a health policy change. Glob Health.

[27] Mensah George A, Fuster V (2021). Race, Ethnicity, and Cardiovascular Disease. J Am Coll Cardiol.

[28] Owusu-Dabo E, Lewis S, McNeill A, Gilmore A, Britton J (2009). Smoking uptake and prevalence in Ghana. Tob Control.

[29] Nketiah-Amponsah E, Afful-Mensah G, Ampaw S (2018). Determinants of cigarette smoking and smoking intensity among adult males in Ghana. BMC Public Health.

[30] Owusu-Dabo E, Lewis S, McNeill A, Anderson S, Gilmore A, Britton J (2009). Smoking in Ghana: a review of tobacco industry activity. Tob Control.

[31] Afrifa–Anane E, Agyemang C, Codjoe SNA, Ogedegbe G, de-Graft Aikins A. The association of physical activity, body mass index and the blood pressure levels among urban poor youth in Accra, Ghana. BMC Public Health 2015;15(1):269. 10.1186/s12889-015-1546-3 PMC4376361 25881047

[32] Tuakli-Wosornu Y, Rowan M, Gittelsohn J (2014). Perceptions of physical activity, activity preferences and health among a group of adult women in urban Ghana: a pilot study. Ghana Med J.

[33] Nti CA, Arthur D, Opare-Obisaw C (2016). Relationship between dietary practices, physical activity and body mass indices of type 2 diabetics attending a clinic in Accra, Ghana. J Public Health Epidemiol.

[34] Osei-Yeboah J, Owiredu W, Norgbe G (2018). Physical activity pattern and its association with glycaemic and blood pressure control among people living with diabetes (PLWD) in the Ho municipality, Ghana. Ethiop J Health Sci..

[35] Nyakotey DA, Ananga AS, Apprey C (2020). Assessing physical activity, nutrient intake and obesity in middle-aged adults in Akuse, Lower Manya Krobo. Ghana. J Health Res..

[36] Mensah D, Aryeetey R, Oyebode O (2022). Evidence on physical activity and sedentary behaviour in Ghana: A rapid scoping review. Afr J Food Agric Nutr Dev.

[37] Konkor I, Dogoli MA, Kuuire V, Wilson K (2021). Examining the Relationship Between Occupational Physical Activity and Hypertension Status: Evidence from the Ghana WHO Study on Global Ageing and Adult Health. Ann Work Exp Health.

[38] De Bruijn A. Alcohol marketing practices in Africa: findings from the Gambia, Ghana, Nigeria and Uganda. 2011.

[39] Mensah NA, Sanuade OA, Baatiema L (2022). Perceptions of community members on contextual factors driving cardiovascular disease behavioural risk in Ghana: a qualitative study. BMC Public Health.

[40] Fernández-Solà J (2015). Cardiovascular risks and benefits of moderate and heavy alcohol consumption. Nat Rev Cardiol.

[41] Krittanawong C, Isath A, Rosenson RS (2022). Alcohol Consumption and Cardiovascular Health. Am J Med.

[42] Arora M, ElSayed A, Beger B (2022). The impact of alcohol consumption on cardiovascular health: myths and measures. Glob Heart.

[43] Marcus GM, Vittinghoff E, Whitman IR (2021). Acute consumption of alcohol and discrete atrial fibrillation events. Ann Intern Med.

[44] Olutobi Adekunle S, Mawuli Komla K, Raphael Baffour A, Paapa Yaw A, Charles A, Ama de-Graft A. Lay knowledge of cardiovascular disease and risk factors in three communities in Accra, Ghana: a cross-sectional survey. BMJ Open 2021;11(12):e049451. 10.1136/bmjopen-2021-049451 PMC8671941 34907046

